# Confounding Effect of Undergraduate Semester–Driven “Academic" Internet Searches on the Ability to Detect True Disease Seasonality in Google Trends Data: Fourier Filter Method Development and Demonstration

**DOI:** 10.2196/34464

**Published:** 2022-07-19

**Authors:** Timber Gillis, Scott Garrison

**Affiliations:** 1 Department of Family Medicine University of Alberta Edmonton, AB Canada

**Keywords:** Google Trends, seasonality, Fast Fourier transform, FFT, pathogenic bacteria, depression, Google search, Google, health information, health information seeking, internet search

## Abstract

**Background:**

Internet search volume for medical information, as tracked by Google Trends, has been used to demonstrate unexpected seasonality in the symptom burden of a variety of medical conditions. However, when more technical medical language is used (eg, diagnoses), we believe that this technique is confounded by the cyclic, school year–driven internet search patterns of health care students.

**Objective:**

This study aimed to (1) demonstrate that artificial “academic cycling” of Google Trends’ search volume is present in many health care terms, (2) demonstrate how signal processing techniques can be used to filter academic cycling out of Google Trends data, and (3) apply this filtering technique to some clinically relevant examples.

**Methods:**

We obtained the Google Trends search volume data for a variety of academic terms demonstrating strong academic cycling and used a Fourier analysis technique to (1) identify the frequency domain fingerprint of this modulating pattern in one particularly strong example, and (2) filter that pattern out of the original data. After this illustrative example, we then applied the same filtering technique to internet searches for information on 3 medical conditions believed to have true seasonal modulation (myocardial infarction, hypertension, and depression), and all bacterial genus terms within a common medical microbiology textbook.

**Results:**

Academic cycling explains much of the seasonal variation in internet search volume for many technically oriented search terms, including the bacterial genus term [“Staphylococcus”], for which academic cycling explained 73.8% of the variability in search volume (using the squared Spearman rank correlation coefficient, *P*<.001). Of the 56 bacterial genus terms examined, 6 displayed sufficiently strong seasonality to warrant further examination post filtering. This included (1) [“Aeromonas” + “Plesiomonas”] (nosocomial infections that were searched for more frequently during the summer), (2) [“Ehrlichia”] (a tick-borne pathogen that was searched for more frequently during late spring), (3) [“Moraxella”] and [“Haemophilus”] (respiratory infections that were searched for more frequently during late winter), (4) [“Legionella”] (searched for more frequently during midsummer), and (5) [“Vibrio”] (which spiked for 2 months during midsummer). The terms [“myocardial infarction”] and [“hypertension”] lacked any obvious seasonal cycling after filtering, whereas [“depression”] maintained an annual cycling pattern.

**Conclusions:**

Although it is reasonable to search for seasonal modulation of medical conditions using Google Trends’ internet search volume and lay-appropriate search terms, the variation in more technical search terms may be driven by health care students whose search frequency varies with the academic school year. When this is the case, using Fourier analysis to filter out academic cycling is a potential means to establish whether additional seasonality is present.

## Introduction

### Google Trends and Disease Seasonality

Google Trends is an open access portal that allows researchers to explore how the public’s quest for information on specific topics varies with time. The data made available by Google Trends is the “volume” (number) of searches for a specific search term entered by the public into the Google search engine per unit time (eg, per week), provided as a percentage of the highest search volume for that term over the period of interest (eg, last 5 years). The data are anonymous and collated geographically, and, given the public use of Google to search for health information [1,2], has been used to establish unexpected seasonality in the symptom burden or incidence of a variety of chronic conditions [3-5]. To describe such population-level investigations of disease processes using web-based data sources, Eysenbach [6] has coined the term “infodemiology.”

“Seasonality” in symptom burden refers to an annual periodicity, or modulation, in some measurable aspect of those symptoms. Much of this modulation may result from seasonal variation in environmental factors that convey the risk of disease. Respiratory viral illnesses are one of the best examples of this [7]. Humidity, temperature, and wind speed are all seasonally modulated, and each factor influences the spread of air-borne pathogens [8]. Mammals additionally have some seasonal modulation of their physiology (eg, body weight, fur thickness, and estrus). While this is not commonly thought of for humans, some studies suggest that even our physiology has some seasonality. Examples of this include higher long-bone growth in children during summer, retention of extracellular water starting in spring, continuing into the summer for patients on dialysis and increased immune system reactivity during winter [9-11]. Outside of infectious diseases, seasonality has also been observed in depression, cardiovascular disease, and overall mortality [12-14]. Recognizing and trying to understand the driving forces behind disease seasonality helps deliver insights that might lead to more effective prevention or treatment of seasonally modulated conditions.

Google Trends has become a popular tool for investigation of disease seasonality. An early use in this area was rapid real-time surveillance of influenza-like illness [15], something that continues to be worked on to augment conventional public health surveillance measures [16]. Others have sought to uncover unexpected seasonality in common conditions such as nocturnal leg cramps, ankle swelling, dental carries, and various mental health disorders [4,5,17-19]. However, a variety of things can confound the use of big data sources such as Google Trends search volume for health information as a proxy for symptom burden [20]. Search terms, for instance, might have dual meanings. Shingles is a disease, but they are also roofing tiles, whose use and related searches might be seasonal in Northern (snow experiencing) climates. Medical conditions can also be more or less newsworthy (eg, when celebrities are involved), and news coverage can sometimes drive search volume more than personal experience with the condition [21]. Influenza surveillance, for instance, has been inconsistent in its predictive ability when compared to hospital-based viral detection [22].

**Figure 1:**
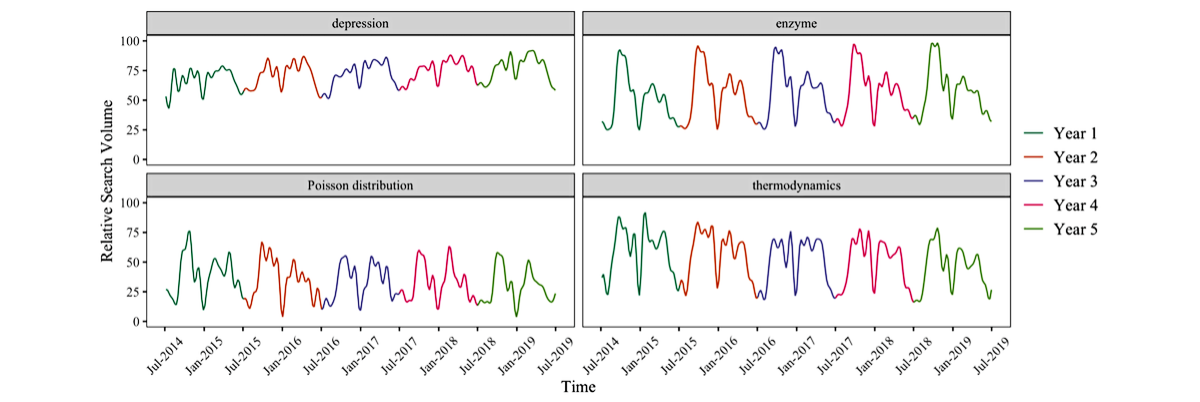
Google Trends search volume for terms with strong academic cycling in the 5 years prior to onset of the COVID-19 pandemic. Searches are limited to the United States, and each color represents a period of 1 year. A high-frequency filter has been applied to remove fluctuations with a period less than 5 weeks (this smooths the curve and eliminates current event driven search volume spikes that last less than 2.5 weeks).

In our use of Google Trends to explore disease seasonality, we have come across an important potential confounder, which has yet to be described. This confounder is the searches for health information carried out by students who are taking courses at the undergraduate level. Such searches can be expected to be low in volume during the summer and winter break (in most countries) and high in volume during the final examination season. We have repeatedly observed such a biphasic seasonal pattern, which we will refer to as “academic cycling,” in many academic-oriented search terms (ie, fairly technical terms that are less commonly used in lay conversation such as proper diagnoses). Such academic cycling spans all fields of study. Some examples from health care, mathematics, and physics are shown in Figure 1. This same academic cycling pattern is clearly present in some of the infodemiology literature, but, even when it appears to be the main driver of the variation in search volume, it is either not acknowledged as such or not accounted for when its presence is recognized [18,23,24]. In this study, we (1) used the fast Fourier transform (FFT) on Google Trends search volume data with strong academic cycling, (2) identified the frequency domain pattern of that academic cycling, (3) searched for and removed that pattern from the frequency domain of search terms where seasonality is of clinical interest, and (4) recreated the time series data for the terms of clinical interest, with the academic cycling component removed. In so doing, we seek to empower researchers with strategies to investigate whether the seasonal trend they see in their Google Trends data is true, disease-related seasonality, or merely a confounding search pattern introduced by academic, school year–driven search volume.

### Fourier Analysis and Filtering

One of the pillars of signal processing is the recognition that time-series data can be represented as the sum of many different sinusoidal waves, each with its own amplitude and phase difference. FFT is a software tool that does just that, representing a given time series (such as our 5-year Google Trends search volume) in the “frequency domain,” by showing what sinusoidal waves would need to be added together to produce the same curve [25]. FFT also lets users go backwards from the frequency domain representation and recreate the time series again (the “inverse FFT”). The advantage of this frequency domain representation is that we can think of our data as having a variety of driving forces and, if the frequencies of those driving forces are unique and can be identified, we can potentially remove them in the frequency domain and put the time series back again without the contribution of the unwanted component. A simple example of this, if one is listening to the radio, would be removing high- or low-frequency “noise” from the radio waves to hear voices more clearly. A more complex use of the same technique might be adding or removing an antipiracy frequency domain watermark from a piece of music or an image [26]. FFT has previously been applied to Google Trends data in order to identify the dominant frequency in time series data describing urinary tract infections and chronic lifestyle diseases [27,28].

## Methods

### Overview

We first demonstrated our filtering process in detail using the term [“thermodynamics”], which was chosen because of its strong academic cycling and helped each step to be visualized. The initial step involved preprocessing of the Google Trends data before FFT could be applied and involved shifting the time-series data down by subtracting the mean value. The resulting transformed data had the same shape as the original time series, but the data were now represented by positive and negative numbers that had a mean value of 0. Although not strictly necessary, we also chose to filter out high-frequency “noise” with FFT to make patterns more visible to the naked eye. These 2 preprocessing steps were applied to both the term of interest and to the control terms that represent the academic cycling that we wish to remove. We then identified how much of the academic cycling component was present in the term of interest by using a least squares regression analysis, subtracted that component in the frequency domain, and recreated the time series with inverse FFT. Following this demonstration, we applied the same technique to a selection of clinically relevant examples.

### Google Trends Data Collection

Google Trends time series data are freely downloadable and presented as the relative search volume (RSV) for the specified search terms per unit time (month, week, day, or hour). An RSV of 0 indicates little to no search volume, and an RSV of 100 indicates the highest volume for that term in the period of interest. We used weekly data for the 5-year period from July 3, 2016, to June 30, 2021. We restricted our analysis to the United States since it was the country with the largest internet search volume and since a single geographic region was needed for most residents to have a shared experience of the changing of the seasons and school year. Our 5-year window was selected to capture 5 full academic years. Although Google Trends provides the option of having search terms represent “topics” (in which case Google Trends aggregates a variety of searches they feel capture the same topic area), this option is not available for all search terms. Hence, for consistency, unless otherwise indicated, we did not use the “topic” search feature. Our search term nomenclature is in accordance with previous literature [29].

### “Fingerprint” Frequency Filtering

#### Overview

**Figure 2:**
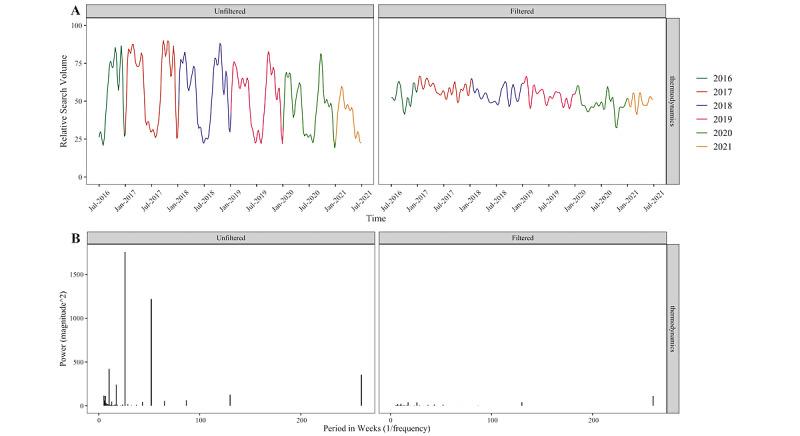
(A) Time series representation of [“thermodynamics”] Google Trends data both before and after removal of academic cycling; color indicates a calendar year. (B) Frequency domain representations of the same time series. Each frequency domain spike is the amplitude of the sinusoids that would need to be combined to produce the time series shown.

Our frequency filtering program was built using R (version 4.0.2) within the RStudio interactive development environment (version 1.4.1106). The process for filtering out academic cycling, every time it was applied, used the following steps. We will illustrate each step using the example term [“thermodynamics”], which displays strong academic cycling. When we refer to the time domain, we mean how the data look as a time series (ie, the way Google Trends initially presents the data in their web browser). When we refer to the frequency domain, we mean the way the data are visualized using the FFT, which is as a series of spikes showing how much of each frequency is present in the data for all of the sinusoids that would need to be combined to create it (Figure 2). Our filtering process involved the following 7 steps.

#### Transformation

We first shifted and scaled the data such that it moved up and down around a mean value of 0 using the following formula:

Transformed RSV = [RSV – mean(RSV)]/mean(RSV)

Once filtering was complete, we applied this transformation in reverse to return to the original scaling.

#### High-Frequency Filtering

Assuming that most high-frequency fluctuations in search volume (ie, sudden changes) are not biologically driven [21], we removed frequencies with a period less than 5 weeks. This effectively removed spikes in search volume, which rose and fell in less than 2.5 weeks, a period we felt would cover most search volume surges triggered by sporadic events or media reports. The smoothing effect of this high-frequency filtering on the term [“thermodynamics”] is shown in Multimedia Appendix 1.

#### Converting to the Frequency Domain

After high-frequency filtering, we applied FFT as natively encoded in R [30] to produce the frequency domain representation shown in Figure 2 (which exhibits major frequency components at 52 weeks and 26 weeks). This representation, however, is a simplification that only shows the amplitude of the frequency component. It is also necessary to know the phase of the sinusoids with those frequencies. Numerically, FFT represents each frequency component with 2 numbers (a “real” and “imaginary” component) that define amplitude and the phase of each sinusoid in the same way that x and y coordinates could be used to define the length and position of the tip of a second hand on a clock’s face.

#### Selection of Control Terms (Terms With Strong Academic Cycling)

The academic cycling pattern that we want to filter out could look different for different disciplines considering the school year and that examination schedules could differ. As such, we chose different control terms for our “thermodynamics” example than we did for our medically relevant examples (choosing [“binomial” + “integral” + “derivative”] as control terms for [“thermodynamics”] and [“gram stain” + “gram positive” + “gram negative”] as control terms for biomedical searches). In the Google Trends browser, using a “+” sign means “or”; that is, [“cat” + “dog”] would count any Google search in which the words “cat” or “dog” were included in the search phrase entered by the user.

#### Identification of the Frequency Domain “Academic Fingerprint” to Be Removed

Similar to our search terms of interest (“thermodynamics” in this example), the search volume for the control terms (ie, [“binomial” + “integral” + “derivative”]) also underwent the first 3 aforementioned steps. The frequency domain pattern of spikes for the control term is the “fingerprint” we intend to filter out of the data for our terms of interest.

#### Determining How Much of the Academic Fingerprint Frequencies to Remove

To best estimate how much of the academic fingerprint was present in a signal, we used a sum of squares minimization approach using the optimize algorithm in R. That is, we took the frequency domain representations of both the term of interest and the control term, and scaled the control term components by an amount k, such that the sum of the squared differences in frequency components between term of interest and control was minimized (note that as shown in Textbox 1, this used the sum of the squared differences of each real and imaginary component and not just the amplitudes). For terms that do not display academic cycling, k was close to 0. For terms with a high degree of academic cycling, k was closer to 1. For our example term [“thermodynamics”], the scaling coefficient (k) for the control term [“binomial” + “integral” + “derivative”] was 0.8663. To remove the academic cycling component, we simply subtracted the scaled frequency components of the control from the same components in the term of interest.

**Scaling approach: minimize algorithm in R minimizes the sum of squared differences (SS2) for the scaling coefficient as represented by “k.”**

SS^2^ = Σ(Real Test – Real Control*k)^2^ + (Imaginary Test – Imaginary Control*k)^2^)

#### Recreating the Time Series Without the Academic Cycling Component

The resultant filtered Fourier coefficients were back-transformed to the time domain using the inverse FFT algorithm, which is part of the same native R function. This allowed us to visualize the time series without the academic cycling, which appears to be eliminated in the “thermodynamics” example (Figure 2).

### Selection of Clinically Relevant Terms to Explore

#### Pathogenic Bacteria

The genus names of pathogenic organisms could be searched for by both patients and providers, who encounter the organism in the usual course of care, and by students learning about such organisms during their training. It is also possible that the abundance of these organisms, their vectors, or the environments in which they are most easily transmitted undergo seasonal modulation. As such, we identified and analyzed 58 pathogenic bacterial genus terms discussed in a common medical microbiology textbook [31]. The genus term [“Bacillus”] was not used as it has a separate meaning in terms of bacterial morphology more generally. After data processing, we also chose to combine the terms [“Aeromonas” + “Plesiomonas”], recognizing both as water-borne pathogens that shared a common taxonomic identity in the past [32]. Recognizing that the search volume for many of these terms would be low, and hence the time series could appear too “noisy” to visibly observe larger trends, we also averaged the Google Trends data for each genus term together to average out random fluctuations and demonstrate whether academic cycling was indeed present in these terms.

#### Conditions Believed to Have Some Seasonality

We also applied our filtering technique to 3 conditions that appeared to have academic cycling and for which previous observational evidence suggests some seasonal modulation; these include depression, myocardial infarction, and hypertension [12,13,33].

### Statistical and Graphical Analyses

Post filtering, for bacterial genus terms, we selected the 6 terms (top 10%) with the strongest annual cycling component (ie, genus names with the highest amplitude frequency domain peaks at 52 weeks) and displayed them graphically. To do this, since these terms generally had a low search volume, and hence a relatively high amount of noise (ie, more seemingly random fluctuations), we graphed the average monthly volume to help average out random fluctuations and make any annual patterns more visible. In order to demonstrate how much the academic searches were driving the search volume for bacterial genus terms, we also calculated the squared Spearman rank correlation coefficient between the time series for each bacterial term and the time series for our control term (ie, [“gram stain” + “gram positive” + “gram negative”]). The squared Spearman rank correlation coefficient was used to estimate the amount of variation in the test data set, which was explained by the variation in the control.

## Results

### Overview

Our filtering technique successfully removed academic cycling from a wide variety of Google Trends data where it is evident. Although the terms [“depression”], [“hypertension”], and [“myocardial infarction”] all had annual cycling prefiltering, this was only evident in searches for [“depression”] once academic cycling was removed. Of 56 pathogenic bacterial genus names, largely because of low search volumes, only 5 displayed substantial annual cycling prefiltering ([“Clostridium”], [“Escherichia”], [“Mycobacterium”], [“Staphylococcus”], and [“Streptococcus”]), and none of these 5 genus names displayed seasonality after academic cycling was removed. After filtering all genus terms, 10% of them with the strongest seasonality (ie, strongest 1-year periodicity in the frequency domain) were [“Aeromonas” + “Plesiomonas”], [“Moraxella”], [“Haemophilus”], [“Ehrlichia”], [“Legionella”], and [“Vibrio”], each of which had search volume peaks consistent with what the clinical literature would predict.

### Pathogenic Bacteria

**Figure 3:**
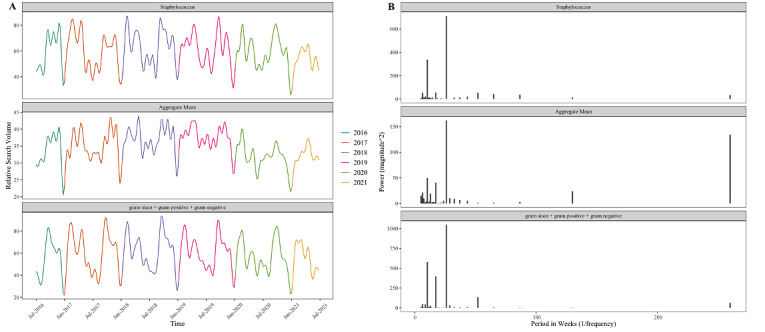
(A) High-frequency filtered Google Trends Internet search volumes for [“Staphylococcus”], the aggregate mean of 56 pathogenic bacterial genus term data (excluding [“Staphylococcus"]), and the [“gram stain” + “gram positive” + “gram negative”] control term used to identify academic cycling in such terms; color indicates a calendar year. (B) The frequency domain representation of the same time series, showing the amplitude of each sinusoid that would need to be summed to obtain the original signal.

Owing to the relatively low search volume, few of our 56 bacterial genus terms displayed obvious academic cycling, with only 5 having a squared Spearman rank correlation coefficient of ≥0.5 with their corresponding control term. Academic cycling was clearly present, however, when the bacterial genus terms were averaged together and in the term [“Staphylococcus”] (Figure 3). Academic cycling explained three-quarters of the variation in the search volume for “Staphylococcus” (ie, *R*^2^=0.74), and half of the variation in our aggregate of 56 other bacterial terms (*R*^2^=0.55).

**Figure 4:**
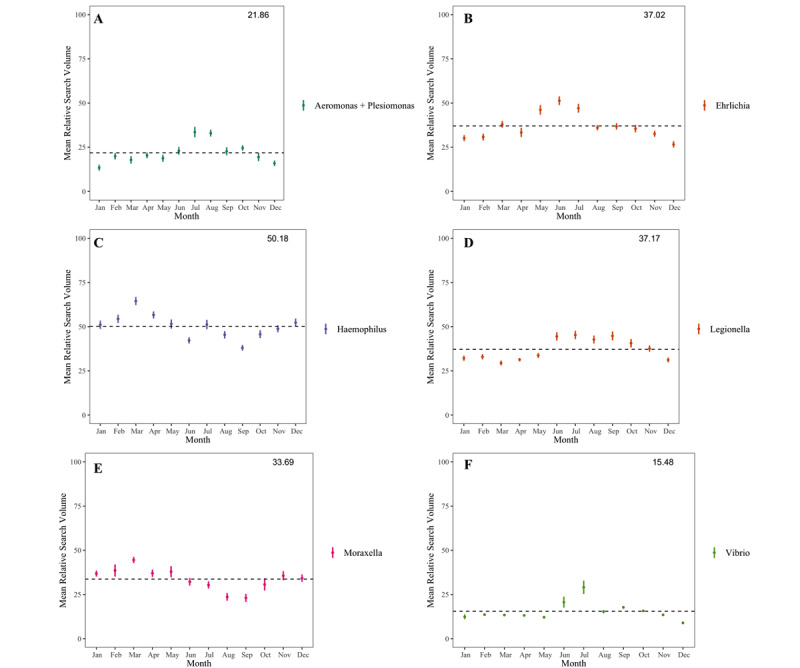
Google Trends internet relative search volume for various pathogenic bacteria, filtered to remove academic cycling, and averaged for each month over a 5-year span from July 3, 2016, to June 30, 2021. (A) [“Aeromonas” + “Plesiomonas”] (combined out of convenience owing to similar reservoirs, similar modes of infection, and historically common taxonomy). (B) [“Ehrlichia”]. (C) [“Haemophilus”]. (D) [“Legionella”]. (E) [“Moraxella”]. (F) [“Vibrio”]. The dotted line is the mean search volume across all 261 data points that are available for averaging. Numbers being averaged are the weekly search volume, obtained as a percentage value of the maximum weekly search volume for that term over the 5-year period.

**Figure 5:**
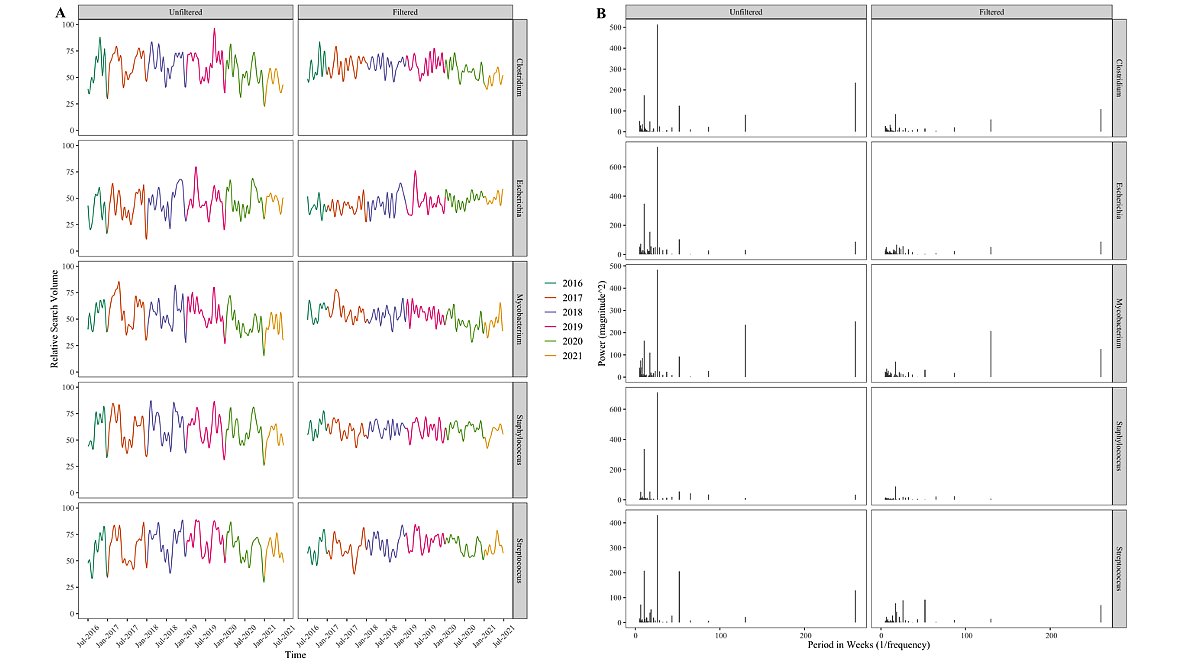
Google Trends internet relative search volume before and after filtering out academic cycling for the terms [“Clostridium”], [“Escherichia”], [“Mycobacterium”], [“Staphylococcus”], and [“Streptococcus”]. (A) These terms in the time domain. (B) The same terms in the frequency domain after applying the fast Fourier transform tool.

The top 10% of genus terms with the most annual cycling (ie, highest 52-week frequency domain peaks) after filtering out academic cycling are shown in Figure 4. [“Aeromonas” + “Plesiomonas”] searches increased during midsummer, [“Moraxella”] and [“Haemophilus”] searches increased during late winter, and “Ehrlichia” search volume spiked in late spring. [“Legionella”] searches had a slow, sustained peak throughout the summer months and during early fall, and [“Vibrio”] searches had a sharp spike during midsummer. All of these had no visible academic cycling and were essentially unaffected by our filter as demonstrated in Multimedia Appendix 1. Only 5 bacterial genus terms had obvious academic cycling, as demonstrated by a squared Spearman rank correlation coefficient of ≥0.5 for comparison with our control terms. These terms were [“Clostridium”], [“Escherichia”], [“Mycobacterium”], [“Staphylococcus”], and [“Streptococcus”], none of which displayed seasonality after filtering (Figure 5).

### Depression, Hypertension, and Myocardial Infarction

**Figure 6:**
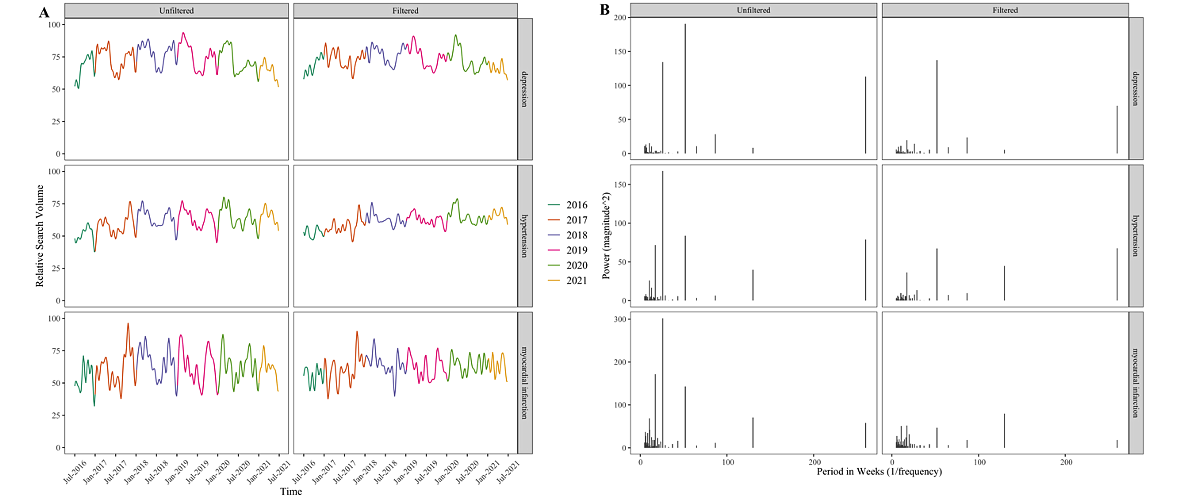
Google Trends internet relative search volume before and after filtering out academic cycling for the terms [“depression”], [“hypertension”], and [“myocardial infarction”]. (A) These terms in the time domain. (B) The same terms in the frequency domain after applying the fast Fourier transform tool.

Academic cycling is evident in searches for information on all 3 of these common conditions (Figure 6). However, after filtering, only [“depression”] displays what appears to be a strong seasonal pattern in the time domain (corresponding to a dominant 52-week peak in the frequency domain), with searches peaking during winter. The terms [“hypertension”] and [“myocardial infarction”] have small peaks at 52 weeks. This could represent a lesser degree of seasonality or perhaps some residual academic cycling that we failed to remove.

## Discussion

Biphasic academic cycling is commonly seen in Google Trends data when technical search terms are used. When this is the case, it can potentially be filtered out using FFT and an appropriate control. Although initially confounded by academic cycling, true seasonality in the public’s searches for information on depression seems to be present. It is less obvious that seasonality is present in searches for information on myocardial infarction and hypertension. Seasonality is also present in searches for information on a variety of pathogenic bacteria.

Biphasic academic cycling patterns are clearly present in some published Google Trends data, but to date, those patterns have been overlooked or given other interpretations. This includes an exploration of the influence of public health campaigns on searches for information on marijuana use, colorectal cancer, and HIV [23]. The search volume for cannabis use in Canada followed a clear biphasic pattern, peaking in the winter and fall, followed by a summer trough. The same is true for an exploration of worldwide searches for information on osteoporosis, where recognizing the academic search pattern, and interpreting it in the light of when school terms start and end in different countries, might have provided an alternate explanation for the observed seasonality of searches and the observed differences between countries [24]. Academic cycling is also clearly present in an exploration of searches for information about mental health conditions [18]. The authors acknowledged the potential for academic searches to confound their findings but considered that its effect would have been negligible.

The months during which we observed higher interest in internet searches on specific bacterial pathogens are consistent with those reported in the microbiology literature. In Hungary, cases of *Plesiomonas* and *Aeromonas* (water-borne pathogens) have been shown to peak between May and September [34]. In the United States, human Ehrlichiosis due to *Ehrlichia* (a tick-borne pathogen) peak in June and July [35]. In the United States and Belgium*, Legionella* respiratory infections rise in summer and autumn [36,37]. In Japan, *Moraxella* respiratory infections are more common in winter [38]. In patients with cystic fibrosis, *Haemophilus* respiratory infections peak in February and March [39]. Furthermore, in the United States, noncholera *Vibrio* gastroenteritis peaks in the summer [40]. Google Trends data have been used to identify the seasonality of searches for antibiotics and probiotics in general (both of which peak in winter), [41] and for tracking and real-time surveillance for viral infections such as influenza [15,16], but we are unaware of it having been used to track the bacterial pathogens we report here.

We chose [“depression”], [“hypertension”], and [“myocardial infarction”] as terms to explore because each has both academic cycling in Google Trends data and epidemiologic evidence of seasonal modulation. Depression and myocardial infarctions have been shown to be more common in winter [12,13], and blood pressure is higher at the same time [33]. This includes Google Trends data showing more searches for [“depression”] in winter in the northern hemisphere [19]. Although our analyses only found obvious seasonality in searches for depression, Google Trends has limitations when it comes to detecting seasonality. If the bulk of internet searches for information on myocardial infarction are not driven by clinical events, seasonality may not be evident. We also assumed that hypertension diagnoses would also be more common in winter because blood pressure in normotensive individuals is higher in winter. This may not be the case. Conceivably, individuals with hypertension might display less seasonal variation in blood pressure readings than do normotensive individuals. It is also possible that the small 52-week peaks that we observed could have resulted from averaging Google Trends data by month, as we did for the low search volume bacterial terms. This has been done by other investigators exploring [“hypertension”] in Google Trends data from Poland, Australia, and the United States [42]. In each case, a winter peak was demonstrated, with a dip in December that was attributed to people possibly being less concerned about their health during Christmas, a dip we would instead attribute to academic cycling.

Our filtering technique is limited by our ability to use an appropriate control. If the shape of the academic cycling in our control term does not match that of the term of interest, its removal would be imperfect or would introduce other seemingly seasonal components. We chose to use the control term [“gram stain” + “gram positive” + “gram negative”] for all our clinical examples because we believed microbiology-related searches would track with health care searching in general. While future researchers could choose to use this same control term to identify and filter out academic cycling, they may alternatively wish to build control terms that display strong academic cycling, which are more specific to the relevant specialty area. We can also only remove academic cycling when it is obviously present. For lower search volume terms, where there is vast higher-frequency “noise,” our filtering method essentially left the waveform intact. As such, our method of averaging together the search volume on a monthly basis to remove some of the noise, and reinforce the seasonal component, would have also reinforced any academic cycling component that was present.

Google Trends internet search volume is a useful tool for detecting disease seasonality when symptoms, or diagnoses, can be expressed in lay terms that have no alternate meaning. Care should be taken, however, to ensure that any emerging cyclic patterns do not have the biphasic pattern that is highly characteristic of searches driven by the academic school year. This is particularly relevant when researchers use more technical terms, such as proper diagnoses. When this is the case, consideration could be given to using the filtering technique we present here, the R script for which is available in Multimedia Appendix 2. With such an approach, we are able to lessen the confounding influence of academic cycling in Google Trends time-series data and increase the likelihood that any residual cycling might have clinical relevance, perhaps being driven by previously unrecognized seasonality that is inherent in human physiology, in the virulence, abundance or reservoirs of pathogenic organisms, or other socioeconomic or behavioral factors that convey risk of illness. Uncovering such seasonality could open up new understanding of human physiology and disease etiology and new opportunities for disease prevention and treatment.

## Appendix

Multimedia Appendix 1 Supplementary figures.

Multimedia Appendix 2 Open-source R code for our Fourier analysis filtering methodology adapted for reader use.

## Abbreviations

FFT: Fast Fourier transform
RSV: relative search volume
SS2: sum of squared differences
