# Supplementary material for: Confounding Effect of Undergraduate Semester–Driven “Academic" Internet Searches on the Ability to Detect True Disease Seasonality in Google Trends Data: Fourier Filter Method Development and Demonstration
Source: JMIR Infodemiology. 2022 Jul 19;2(2):e34464. doi: 10.2196/34464 (PMC9987186; doi:10.2196/34464)
Supplement: Multimedia Appendix 1 [file infodemiology_v2i2e34464_app1.docx]

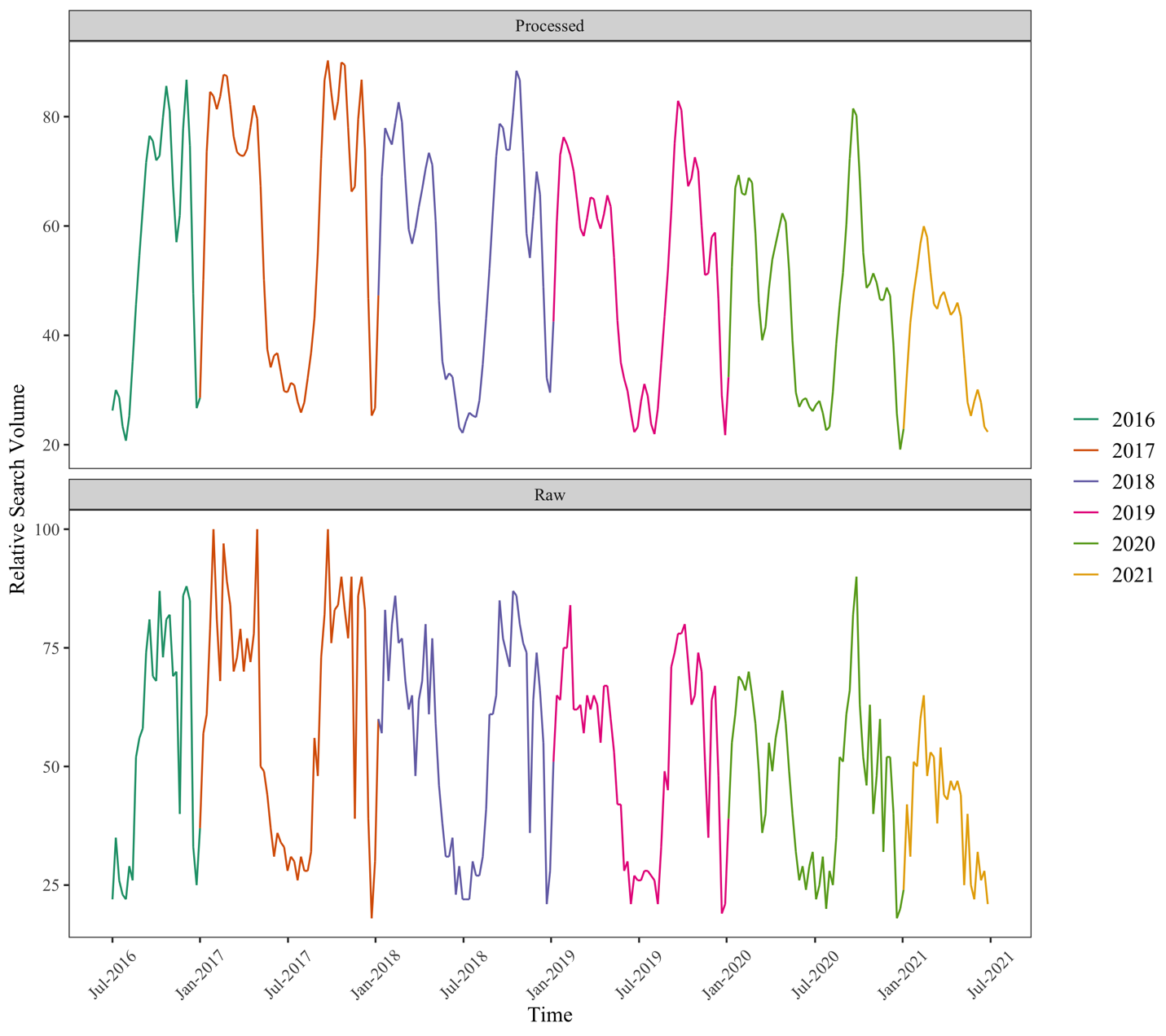


Supplementary Figure 1: [“Thermodynamics”] search term, unfiltered data in the time domain. Comparison between low-pass-filter processed data (Processed) and the raw data obtained from Google Trends (Raw).


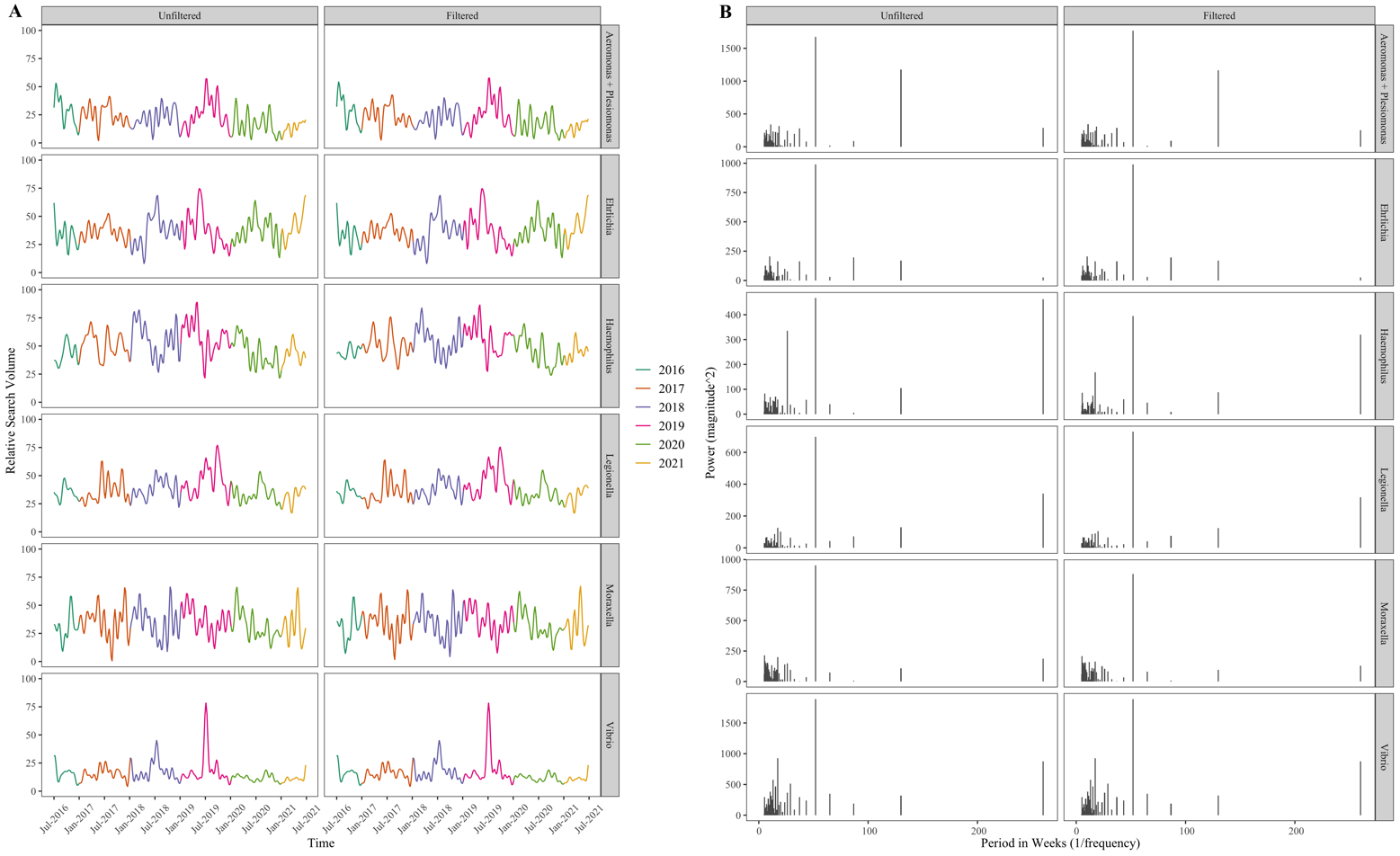


Supplementary Figure 2: A: Google Trends search volume data for [“Aeromonas” + “Plesiomonas”], [“Ehrlichia”], [“Haemophilus”], [“Legionella”], [“Moraxella”] and [“Vibrio”] before and after filtering out academic cycling with the control term [“gram stain” + “gram positive” + “gram negative”]. B: The same time series after fast Fourier transformation, representing the amplitude of the sinusoids that would need to be combined to create the corresponding waveforms.

|  | Aggregate Mean | Control | Staphylococcus |
| --- | --- | --- | --- |
| Aggregate Mean | 1 | 0.4804177 | 0.5482171 |
| Control | 0.4804177 | 1 | 0.7380573 |
| Staphylococcus | 0.5482171 | 0.7380573 | 1 |

Supplementary Table 1: Spearman’s rank correlation table. Cells are rho^2^ where rho^2^ is the proportion of the variation explained by the corresponding term with which it is correlated. All terms were significantly correlated with one another (p < 0.05).

|  | Clostridium | Control | Escherichia | Mycobacterium | Staphylococcus | Streptococcus |
| --- | --- | --- | --- | --- | --- | --- |
| Clostridium | 1 | 0.5721 | 0.4160 | 0.5515 | 0.5997 | 0.5629 |
| Control | 0.5721 | 1 | 0.6222 | 0.5326 | 0.7381 | 0.5513 |
| Escherichia | 0.4160 | 0.6222 | 1 | 0.2840 | 0.5140 | 0.5031 |
| Mycobacterium | 0.5515 | 0.5326 | 0.2840 | 1 | 0.5663 | 0.5127 |
| Staphylococcus | 0.5997 | 0.7381 | 0.5140 | 0.5663 | 1 | 0.6005 |
| Streptococcus | 0.5630 | 0.5513 | 0.5031 | 0.5127 | 0.6005 | 1 |

Supplementary Table 2: Squared Spearman’s rank coefficient table corresponding to the five bacterial genus terms which had a R^2^ value of 0.5 corresponding to the control term ([“gram stain” + “gram positive” + “gram negative”]).
